# Central Sensitization in Spondyloarthritis: Implications for Personalized Medicine

**DOI:** 10.3390/jpm16050252

**Published:** 2026-05-05

**Authors:** Linda Carli, Federico Fattorini, Marco Di Battista, Lorenzo Esti, Cosimo Cigolini, Marta Mosca, Andrea Delle Sedie

**Affiliations:** 1 Rheumatology Unit, Department of Medical and Surgical Specialties, Azienda Ospedaliero-Universitaria Pisana (AOUP), Via Roma 67, 56126 Pisa, Italycosimo.cigolini@gmail.com (C.C.); marta.mosca@unipi.it (M.M.); adellesedie@gmail.com (A.D.S.); 2 Rheumatology Unit, Department of Clinical and Experimental Medicine, University of Pisa, Via Roma 67, 56126 Pisa, Italy; federicofattorini19@gmail.com (F.F.); lorenzo.esti@gmail.com (L.E.)

**Keywords:** Spondyloarthritis, central sensitization, quality of life

## Abstract

**Background**: Central sensitization (CS) has been held responsible for both persistent pain and high disease activity scores in Spondyloarthritis (SpA). The Central Sensitization Inventory (CSI) is a questionnaire used to determine CS frequency: a score of at least 40 is associated with a high likelihood of CS. **Objectives**: To investigate the prevalence of CS in our cohort and its association with clinical characteristics of patients and their quality of life. **Methods**: Adult patients with a diagnosis of Psoriatic Arthritis (PsA) or Axial Spondyloarthritis (AxSpA) who were also classifiable according to ClASsification criteria for Psoriatic Arthritis (CASPAR) and Assessment of SpondyloArthritis international Society (ASAS) criteria respectively, and regularly followed at the SpA outpatient clinic of our Unit were consecutively enrolled from April to November 2023. Their epidemiologic, clinical and clinimetric data were collected, as well as patient-reported outcome measures (PROMs) [CSI, Health Assessment Questionnaire (HAQ), FACIT-Fatigue (FACIT-F), SHORT-FORM 36 (SF-36), and Hospital Anxiety and Depression Scale (HADS)]. Considering the definition of “difficult-to-treat” rheumatoid arthritis, we defined as “multi-failure” those patients who were treated with more than two biologic disease-modifying anti-rheumatic drugs (bDMARDs) with different mechanisms of action. Intergroup comparisons were assessed by using Chi-square, *t*-test and ANOVA. *p*-values < 0.05 were considered significant. **Results**: A total of 100 patients were enrolled, 46 male (46.0%) and 54 female (54.0%), with a mean age of 59.4 ± 9.8 years and a mean disease duration of 14.8 ± 10.1 years; 79 patients (79%) had a diagnosis of PsA and 21 (21%) of AS. Forty-two patients (42.0%) had a CSI score ≥ 40. Significant correlations were found between a CSI score ≥ 40 and female sex (*p* = 0.004), the occurrence of enthesitis (*p* = 0.05), DAPSA-CRP (*p* = 0.02) and ASDAS scores (*p* = 0.03), a multi-failure condition (*p* = 0.01), fibromyalgia (FM) (*p* = 0.004), thyroid disease (*p* = 0.016) and obesity (*p* = 0.047). Regarding PROMs, significant correlations were found between CSI and values of HADS (both anxiety and depression), FACIT-F, HAQ and all the domains of SF-36 (*p*-value < 0.0001). **Conclusions**: Our data confirmed that more than 40% of SpA patients had CSI values ≥ 40 and underlined how CS could widely impair their disease burden. A routinary evaluation of CS and a multifactorial biopsychosocial perspective in the diagnosis and management of chronic pain in patients with SpA could help rheumatologists in improving their quality of care.

## 1. Introduction

Spondyloarthritis (SpA) is a group of inflammatory diseases involving both peripheral and axial joints and extra-articular domains.

Five major subtypes of SpA are recognized: Ankylosing Spondylitis (AS), Reactive Arthritis (ReA), Psoriatic Arthritis (PsA), Arthritis associated with Inflammatory Bowel Disease (SpA-IBD), and Undifferentiated Spondyloarthritis (uSpA) [1]. More recently, a classification into axial and peripheral diseases has been proposed [2].

A regular monitoring of disease activity and a shared decision-making approach are critical for the long-term management of chronic diseases such as SpA [3]. Different kinds of inflammatory involvement in SpA tend to be associated with pain, in particular synovitis, tenosynovitis and enthesitis. A few new targeted therapies have been approved in recent years with good efficacy and safety data that, in a future perspective, could be able to significantly improve the control of disease activity and consequently patients’ quality of care. In this context, the progressive shift toward personalized medicine in rheumatology highlights the need to better characterize the heterogeneous mechanisms underlying pain and disease burden in SpA patients, in order to further tailor therapeutic strategies to each individual patient.

However, it is reasonable to postulate that patients’ pain is not only related to joint or peri-articular structure inflammation, but also to other processes associated with pain perception, in particular neuroinflammatory processes and pain-processing mechanisms, including central sensitization (CS) [4]. The International Association for the Study of Pain (IASP) defined CS as “an increased responsiveness of nociceptive neurons in the central nervous system (CNS) to their normal or subthreshold afferent input”.

Therefore, CS involves multiple CNS dysfunctions, including impaired sensory processing in brain regions involved in acute pain perception, as well as altered activity in nociceptive facilitatory pathways [5].

CS has been held responsible for both persistent pain and high disease activity scores in SpA [6] and has recently been recognized as a potential pathophysiological mechanism underlying a group of chronic pain disorders including fibromyalgia (FM), temporomandibular joint disorder (TMJD), irritable bowel syndrome (IBS), interstitial cystitis, tension-type headache (TTH), chronic low-back pain, chronic neck pain and myofascial pain syndrome [7,8].

To assess the presence of CS, the Central Sensitization Inventory (CSI) questionnaire has been administered to patients with different pathologies as migraine [9], chronic plaque psoriasis [10], irritable bowel syndrome, chronic pain syndromes and inflammatory bowel disease [11]: a score of at least 40 has been associated with a high likelihood of CS [12]. The CSI has proven to be a tool able to reliably determine the presence of CS; moreover, it can be useful for highlighting the presence of CS-associated syndromes [13].

Indeed, the CSI consists of two sections: parts A and B. CSI-A contains 25 items exploring emotional and somatic disorders associated with CS. Each response is scored from 0 to 4, yielding a total score from 0 to 100: a higher score indicates a more severe symptomatology. The second part of the inventory, CSI-B, explores CS Syndromes (CSSs), conditions associated with CS that cannot be precisely defined but that share symptoms, such as restless leg syndrome, chronic fatigue syndrome, FM, TMJDs, migraine/TTH, IBS, multiple chemical sensitivity, whiplash, anxiety/panic attacks and depression [7].

The aim of our study was to investigate the prevalence of CS in our cohort of SpA patients and its association with clinical characteristics of patients and their quality of life (QoL), evaluated with patient-reported outcomes measures (PROMs).

## 2. Materials and Methods

### 2.1. Study Design and Population

Adults attending the SpA outpatient clinic of the Rheumatology Unit, Department of Clinical and Experimental Medicine, University of Pisa (Pisa, Italy), were consecutively recruited for this cross-sectional study. All participants had a confirmed diagnosis of PsA or AS and fulfilled the respective CASPAR (ClASsification criteria for Psoriatic ARthritis) and ASAS (Assessment of SpondyloArthritis international Society) classification criteria. Patients were under regular follow-up at the clinic and were enrolled consecutively between April and November 2023.

### 2.2. Data Collection

The following epidemiological, clinical, and clinimetric variables were recorded: demographic characteristics (age and sex), diagnosis, age at disease onset, initial symptoms, and extra-articular manifestations [Inflammatory Bowel Diseases (IBDs), uveitis, and psoriasis]. Musculoskeletal involvement was assessed in terms of axial and/or peripheral arthritis, entheseal disease, and dactylitis, while disease activity was evaluated using ASDAS-CRP and DAPSA. Comorbid conditions were also documented, including [osteoporosis (OP), osteoarthritis (OA) whether clinically relevant or symptomatic, hypertension, chronic obstructive pulmonary disease (COPD), interstitial lung disease, ischemic heart disease, FM, diabetes, hyperuricemia, thyroid disorders, metabolic syndrome, dyslipidemia, obesity, and psychiatric disorders].

In addition, a comprehensive treatment history was collected, including exposure to non-steroidal anti-inflammatory drugs (NSAIDs), glucocorticoids (GCs), conventional synthetic disease-modifying anti-rheumatic drugs (csDMARDs), biological disease-modifying anti-rheumatic drugs (bDMARDs), and targeted synthetic disease-modifying anti-rheumatic drugs (tsDMARDs). Based on the definition of “difficult-to-treat” rheumatoid arthritis [14] and on recent EULAR recommendations and consensus definitions for difficult-to-manage and treatment-refractory Psoriatic Arthritis [15,16], patients who had received more than two bDMARDs with different mechanisms of action were classified as having “multi-failure SpA”. Patient-reported outcomes (PROMs) included the CSI, Patient Global Assessment (PGA), Health Assessment Questionnaire (HAQ), FACIT-Fatigue (FACIT-F), Short Form-36 (SF-36), Hospital Anxiety and Depression Scale (HADS), and Work Productivity and Activity Impairment Questionnaire (WPAI).

Data were extracted from an institutional clinical database including patients followed at the SpA Clinic of the Rheumatology Unit, University of Pisa, originally established as a broader ethics committee-approved study, from which the present analysis was derived. 

The study population was stratified according to a CSI threshold of 40, which indicates a high probability of central sensitization.

### 2.3. Statistical Analysis

Population characteristics are reported as counts and percentages, mean ± standard deviation, and median (IQR) for categorical variables, normally distributed continuous variables, and non-normally distributed continuous variables, respectively. Between-group comparisons were performed using the Chi-square test, *t*-test, and ANOVA. A *p*-value < 0.05 was considered statistically significant. All analyses were carried out with R software (R Core Team 2025, R 4.5.0 Foundation for Statistical Computing, Vienna, Austria). 

### 2.4. Ethical Considerations

This cross-sectional observational study was conducted in accordance with the Declaration of Helsinki and was approved by the local ethics committee (Comitato Etico di Area Vasta Nord Ovest; approval number 20070, dated 9 September 2021). The approval covers the collection and use of clinical data within an institutional database of patients with SpA followed at the Rheumatology Unit, University of Pisa.

The present study represents a secondary analysis of this collected dataset, focusing on a specific research question within the broader study framework. Written informed consent (Study Promotor: Azienda Ospedaliero Universitaria Pisana, version 1) was obtained from all participants prior to inclusion in the study. Patients who were minors or who did not provide signed informed consent were excluded. In addition, all participants provided consent for the publication of anonymized data.

## 3. Results

A total of 100 patients were enrolled, 54 female (54%), with a mean age of 59.4 ± 9.8 years and a mean disease duration of 14.8 ± 10.1 years; 79 patients (79%) had a diagnosis of PsA and 21 (21%) of AS. Demographic characteristics of patients are reported in Table 1.

Table 2 summarizes the distribution of mean CSI values according to the different therapeutic regimens, including csDMARDs, tsDMARDs, and bDMARDs.

The mean value of CSI was 35.9 ± 16.9; 42 patients (42%) had a CSI score ≥ 40. Demographic, clinical, and clinimetric characteristics of patients with a CSI ≥ or <40 were compared (Table 3).

Among demographic and clinical characteristics, a significant correlation was found between a CSI score ≥ 40 and female sex (*p* = 0.004) and entheseal involvement (*p* = 0.045).

**Figure 1:**
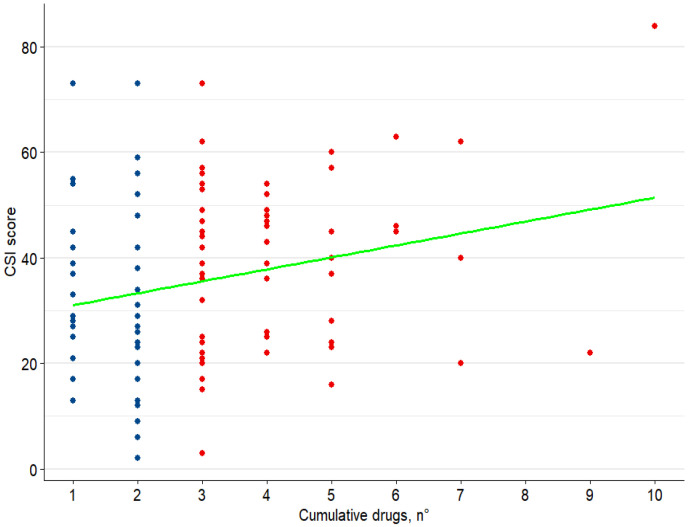
Relationship between CSI score and number of cumulative DMARDs (biological or targeted synthetic). Red dots represent multifailure patients fulfilling the definition of difficult-to-treat (D2T), defined as failure of ≥2 bDMARDs/tsDMARDs with different mechanisms of action; blue dots represent non-D2T patients. The green line indicates the linear regression between cumulative DMARDs and CSI score.

Considering clinimetric indices, SpA patients with a CSI ≥ 40 exhibited significantly higher ASDAS-CRP (*p* = 0.031) and DAPSA (*p* = 0.019) scores than those with a CSI score < 40. Moreover, a multi-failure status was significantly more frequent in patients with a higher CSI; accordingly, in this subgroup, patients had been treated with a higher number of immunosuppressive drugs (see Figure 1).

On the contrary, the two subgroups were comparable in terms of age, diagnosis, disease duration, history of SpA or psoriasis, peripheral arthritis, sacroiliitis, dactylitis, and tenosynovitis.

Regarding comorbidities, FM (*p* = 0.004), thyroid diseases (*p* = 0.016) and obesity (*p* = 0.047) were significantly associated with higher CSI scores (Table 4).

No statistically significant differences in the mean CSI values emerged among the different drug classes.

Regarding PROMs, significant direct correlations were found between the CSI and PGA and scores of HADS (both anxiety and depression), FACIT-F and HAQ, while a significant indirect correlation was observed with all the domains of SF-36 and WPAI presenteeism, work productivity loss and limitation in non-work daily activities (*p* < 0.0001) (Table 5).

Following univariate correlation analysis, a multiple linear regression analysis was conducted. In the multivariate analysis, the CSI score remained significantly associated with multi-failure patients (*p* = 0.02), female sex (*p* = 0.01), HAQ (*p* = 0.005) and PGA (*p* = 0.03).

## 4. Discussion

In a large cross-sectional study on patients with axSpA, van der Kraan and colleagues demonstrated that the CSI could be useful to assess the potential presence of CS both in daily clinical practice and for research purposes [17]. Patients with a CSI ≥ 40 exhibited significantly higher hyperalgesia and increased pain facilitation [8].

### 4.1. Prevalence of CS ≥ 40

The prevalence of CS in inflammatory arthritis is around 31–45% [6], being much higher than that reported in the general population (about 5–15% [7,8]).

Guler et al. assessed CS in patients with different rheumatic diseases using the CSI, highlighting a prevalence of 45% in SpA, 41% in RA, 62% in OA and 94% in FM [8]. In a recent Turkish study, the prevalence of CS in axSpA was confirmed at 41%, similar to that found in Familiar Mediterranean Fever patients [18].

The prevalence of CS in our case cohort appears to be consistent not only with these already mentioned results, but also with some other data from the literature [6,19,20,21,22]. On the contrary, Sariyildiz and colleagues found a prevalence of CS in axSpA patients of around 60% (see also Table 6) [21].

### 4.2. Clinical Association with CSI ≥ 40

The already available data from the literature showed that Salaffi et al. found an association between a CSI ≥ 40 and higher disease activity [20], as also confirmed by Karlibel [6], who also observed an association between CS and female sex. Accordingly, data from Kieskamp [23] showed an association between CS and female sex, enthesitis and comorbidities such as obesity and depression, while in the cross-sectional study by Sariyildiz, CSI score correlated with enthesis involvement and anxiety [21] (see Table 7).

In agreement with Kieskamp [23] and Karlibel [6], our data confirmed the correlation between higher CSI values and female sex. Several studies indicate the existence of a sexual dimorphism in chronic pain, with women showing a greater susceptibility to pain than men in most chronic pain conditions [24]. Despite a comparable control of inflammation, female axSpA patients tend to show higher disease activity and pain scores, with worse QoL outcomes than men [25].

As already demonstrated by Salaffi [20] and Karlibel [6], and also confirmed in our cohort, pain sensitivity and neuropathic-like pain are related to higher disease activity, reported in terms of clinimetric indices (i.e., DAPSA and ASDAS-CRP).

Moreover, we also observed that a “multi-failure” status was more frequently exhibited by patients with a CSI ≥ 40. A remarkable proportion of patients with SpA remain resistant to DMARDs with different mechanisms of action and a recent GRAPPA review [26] showed that the failure to achieve remission in PsA is influenced by persistent inflammatory activity and scarce adherence to treatments. In line with recent EULAR points to consider, difficult-to-manage PsA is a multifactorial condition in which, beyond inflammatory activity, comorbidities, pain amplification mechanisms and patient-related factors may contribute to treatment refractoriness and persistent symptoms [15]. However, the presence of chronic pain due to structural damage or hypersensitisation also seems to reduce the efficacy of different therapies; furthermore, it could distort the estimate of disease activity. Therefore, the association between a CSI ≥ 40 and a multi-failure condition could depend on an insufficient control of pain sensitization, rather than persistent disease activity. From this perspective, the identification of CS may represent an important step toward a more personalized medicine approach, helping clinicians to distinguish inflammatory-driven symptoms from pain amplification mechanisms and to better tailor pharmacological and non-pharmacological interventions.

Newly in agreement with Kieskamp [23], we also found a significant association between obesity and CSI scores [27]. Some already published results showed the relationship between obesity and higher values of both CRP and ASDAS-CRP [28].

In fact, adipose tissue can be considered as an active endocrine organ, excreting adipocytokines or adipokines like TNF-α, which may at least partially explain the proinflammatory state characterizing people with obesity [29]. Moreover, obesity may impair the assessment of swollen joint count, thus further increasing the risk of failing to estimate disease activity in this subgroup of patients [29]. CSI score in our cohort correlated with FM, a frequent comorbidity in patients with SpA, especially in peripheral forms. FM is characterized by chronic widespread pain, fatigue and sleep disturbances; is driven by the effects of chronic pain and inflammation; and could be influenced by patients’ psychoemotional background. It could be another cause of an overestimation of disease activity, thus leading to possibly inappropriate treatment escalation [30].

Interestingly, our results highlighted that thyroid disorders (TDs) seemed to be associated with a higher risk of CS. This relationship is poorly explored in the literature: a possible mechanism of pain generation could be related to an acquired “channelopathy”, involving ion channels, that has been already described in TDs and in FM [31]. The presence of thyroid autoantibodies was associated with more severe FM symptoms, supporting a possible role of thyroid dysfunction in the development of CS [32].

### 4.3. CS, QoL and Work Ability

Functional disability, poor QoL and worse mental health are also related to pain sensitivity and neuropathic-like pain. Persistent pain significantly reduces QoL and, particularly if widespread, it may result in unnecessary anti-rheumatic treatment and higher levels of emotional distress, such as depression and anxiety [33].

The association between poorer QoL outcomes and CS was highlighted in our SpA cohort, in line with the findings in the literature.

Indeed, Salaffi [20], Karlibel [6] and colleagues observed an association among CS, a worse functional ability and a worse QoL. Moreover, Karlibel also found a close relationship between CS severity and sleep disorders [6].

Accordingly, our results highlighted that CS was able to widely impair both mental and physical components in QoL outcomes.

Finally, SpA affects the working ability of patients, increasing absenteeism and work productivity loss, with possible psychosocial repercussions. Tekaya and colleagues used the WPAI to evaluate CS impact on work ability, highlighting that high CSI scores were correlated with work activity limitations [34]. Similarly, we found correlations among higher CSI scores and presenteeism, work productivity loss and reduced ability to perform non-work daily activities, as assessed by the WPAI.

Our work is one of the few that evaluate CS in Italian SpA patients, also exploring the relationship with work productivity.

These data suggest that CS might significantly worsen the disease’s burden on patients with SpA; therefore, it is recommended that rheumatologists s regularly assess its occurrence in clinical practice, thus aiming to improve patients’ quality of care. In addition, incorporating CS assessment into routine evaluation may contribute to a more individualized management strategy, consistent with the principles of personalized medicine, allowing clinicians to better stratify patients according to pain mechanisms and optimize therapeutic decisions.

In our opinion, this work analyses the impact of CS on SpA patients from a very wide perspective, starting from epidemiologic characteristics, passing by clinical features and finishing with QoL outcomes, and is able to highlight some not already explored relationships among CSI results and SpA phenotypes.

The major limitation of our study was a relatively small sample size. Longitudinal studies including larger patient cohorts are needed to further explore this issue.

Finally, we collected treatment duration data, enabling evaluation of the effects of specific drugs on pain and, consequently, on CSI. However, we did not observe any significant differences among mean CSI values and the drug class administered. This result may be associated with the relatively low number of patients that comprised the different drug class subgroups, thus strengthening the need to increase the sample of analyzed patients.

Several methodological considerations should be taken into account when interpreting our findings. The observed correlations between the CSI and other clinical scores should be interpreted with caution, as some degree of overlap exists among the evaluated instruments, particularly with regard to domains such as pain and fatigue. Moreover, key disease activity indices, such as DAPSA and ASDAS, include PROMs (e.g., pain and global assessment) that are inherently influenced by central sensitization, raising the possibility of partial circularity. Therefore, part of the association between the CSI and disease activity may reflect measurement overlap rather than a fully independent relationship.

In addition, the study relies exclusively on the CSI, which is a screening tool rather than a formal diagnostic instrument for central sensitization or nociplastic pain. As such, the CSI may capture broader dimensions, including psychological distress, fatigue, and general symptom burden, thus posing potential limitations in terms of construct validity. Nevertheless, it remains a widely used and practical instrument that provides clinically relevant insight into symptom clusters associated with altered pain processing. Furthermore, although multivariable analyses were performed, residual confounding cannot be excluded, as some relevant factors strongly associated with the CSI—such as coexisting FM, psychological distress, and obesity—could not be fully addressed because of data limitations.

From a clinical perspective, despite these limitations, the consistent associations observed support the hypothesis that central sensitization contributes to the overall disease burden, providing insights that complement traditional inflammatory markers. These findings have significant implications for patient management: identifying high CSI scores can help clinicians better contextualize persistent symptoms and foster a more individualized, multidimensional treatment approach. This may include adjunctive strategies targeting pain modulation alongside non-pharmacological interventions. Furthermore, acknowledging the role of central sensitization may prevent the unwarranted escalation of immunomodulatory therapies in patients whose symptoms are not primarily driven by active inflammation. Finally, due to its cross-sectional design, this study cannot establish causal relationships between the observed associations. It remains unclear whether central sensitization drives increased disease activity and treatment resistance, or if it arises as a consequence of prolonged disease burden and suboptimal control. Longitudinal studies are therefore warranted to clarify this temporal relationship, better understand the directionality of these associations, and evaluate the impact of CSI-guided management on clinical outcomes.

## 5. Conclusions

CS is frequent among SpA patients, and it seems to be associated with female sex, a higher level of disease activity and a multi-failure status. Furthermore, patients with obesity and patients with FM are at higher risk of developing significant CS; interestingly, TDs seem to significantly favour its onset. Finally, CS has been confirmed as a cause of a wide impairment of QoL outcomes, both physical and psychological, together with a consistent worsening in patients’ work productivity Furthermore, recognizing central sensitization in clinical practice may facilitate more personalized management, integrating inflammatory disease control with targeted pain modulation strategies. In our opinion it is possible to highlight some “key” messages about this issue:

- SpA patients should be regularly screened for CS, to reduce the risk of unwarranted immunosuppression and to optimize the management of their pain within a personalized medicine framework;
- Imaging techniques (namely ultrasound and to a lesser extent MRI) could be of help in the management of patients with higher CSI values, to confirm the presence of disease activity (especially for evaluating enthesitis);
- It is important to adopt a multifactorial biopsychosocial perspective in the diagnosis and management of chronic pain in patients with SpA, aiming at optimizing their quality of care.

## Figures and Tables

**Table 1:** Demographic characteristics of patients. n (%); mean (SD).

Characteristic	N = 100
Sex N (%)	F 54.0 (54.0%)
Age (yrs)	59.4 (9.8)
Diagnosis N (%)	PsA 79 (79.0%)
Disease duration (yrs)	14.8 (10.1)

**Table 2:** Details of therapies performed on patients with average CSI; csDMARDs: MTX, LEF, HCQ, and CyA.

N° Patients		Average CSI
csDMARDs	33	**30.2**
Apremilast	2	25
TNFi	34	28.6
IL-17i	15	33.25
IL-12/23i	1	54
IL-23i	2	33
JAKi	6	42.5
TNFi + MTX	5	14.8
IL17i + MTX	2	26.5

**Table 3:** Comparison of demographic, clinical, and clinimetric data between patients with CSI ≥ 40 and those with CSI < 40.

	CSI Value < 40, N = 58	CSI Value ≥ 40, N = 42	*p*-Value
Female sex	24.0 (41.4%)	29.0 (69.0%)	**0.004**
Age	60.6 (9.1)	57.6 (10.6)	N.S.
AP diagnosis	45.0 (77.6%)	34.0 (80.9%)	
AS diagnosis	13.0 (22.4%)	8.0 (19.1%)	
Disease duration	196.1 (136.6)	153.5(91.5)	N.S.
Family history of SpA	0.0 (0%)	3.0 (7.3%)	N.S.
Family history of psoriasis	4.0 (6.9%)	4.0 (9.8%)	N.S.
Arthritis	36.0 (62.1%)	30.0 (73.2%)	N.S.
Dactylitis	9.0 (15.5%)	2.0 (4.9%)	N.S.
Enthesitis	4.0 (9.8%)	15.0 (25.9%)	**0.045**
Tenosynovitis	11.0 (19.0%)	6.0 (14.6%)	N.S.
Ultrasound synovitis	16.0 (27.6%)	5.0 (12.2%)	N.S.
Ultrasound dactylitis	1.0 (1.7%)	0.0 (0%)	N.S.
Ultrasound enthesitis	4.0 (6.9%)	4.0 (6.9%)	N.S.
Ultrasound tenosynovitis	3.0 (5.2%)	3.0 (7.3%)	N.S.
Sacroiliitis on MRI	16.0 (27.6%)	14.0 (34.1%)	N.S.
Spondylitis on MRI	3.0 (5.2%)	1.0 (2.4%)	N.S.
ASDAS-CRP	0.30 (0.20)	3.02 (0.99)	**0.031**
DAPSA	16.4 (6.7)	6 (5.1)	**0.019**
Multi-failure	28.0 (48.3%)	30.0 (73.2%)	**0.013**
Total of drugs	2.7 (1.6)	3.7 (2.0)	**0.009**

n (%); mean (SD). *p*-value calculated with Pearson’s Chi-squared test, Welch’s Two-Sample *t*-test, and Fischer’s exact test. Bold values indicate statistically significant *p*-values (*p* < 0.05).

**Table 4:** Comparison in comorbidities’ development between patients with CSI ≥ 40 and those with CSI < 40.

	CSI Value ≥ 40, N = 42	CSI Value < 40, N = 58	*p*-Value
**Extra-skeletal manifestations**			
Uveitis	2.0 (4.9%)	4.0 (6.9%)	N.S.
Psoriasis	24.0 (58.5%)	35.0 (60.3%)	N.S.
Inflammatory bowel diseases	1.0 (2.4%)	3.0 (5.2%)	N.S.
**Comorbidities**			
Osteoporosis	7.0 (17.1%)	11.0 (19.0%)	N.S.
Osteoarthritis	19.0 (46.3%)	21.0 (36.2%)	N.S.
Ischemic heart disease	10.0 (25.0%)	12.0 (20.7%)	N.S.
Arterial hypertension	8.0 (19.5%)	17.0 (29.3%)	N.S.
Chronic renal failure	5.0 (12.2%)	3.0 (5.2%)	N.S.
COPD	2.0 (4.9%)	3.0 (5.2%)	N.S.
Interstitial lung disease	1.0 (2.4%)	5.0 (8.6%)	N.S.
BMI > 30	8.0 (19.5%)	3.0 (5.2%)	**0.047**
Thyroid disorders	12.0 (29.3%)	6.0 (10.3%)	**0.016**
Psychiatric disorders	7.0 (17.1%)	4.0 (6.9%)	N.S.
Hyperuricemia	3.0 (7.3%)	3.0 (5.2%)	N.S.
Diabetes mellitus	1.0 (2.4%)	6.0 (10.3%)	N.S.
Dyslipidemia	13.00%	9.0 (15.5%)	N.S.
Metabolic syndrome	4.0 (9.8%)	4.0 (6.9%)	N.S.
Fibromyalgia	15.0 (36.6%)	7.0 (12.1%)	**0.004**
Total comorbidities	3.6 (2.2)	2.7 (1.9)	N.S.

n (%); mean (SD). *p*-value calculated with Pearson’s Chi-squared test, Welch’s Two-Sample *t*-test, and Fischer’s exact test. Bold values indicate statistically significant *p*-values (*p* < 0.05).

**Table 5:** Comparison in PROMs outcomes between patients with CSI ≥ 40 and those with CSI < 40.

	CSI Value < 40, N = 58	CSI Value ≥ 40, N = 42	*p*-Value
PGA	3.7 (2.4)	6.7 (1.6)	**<0.001**
HAQ	0.2 (0.3)	0.8 (0.4)	**<0.001**
FACIT-F	41.4 (7.5)	28.7 (8.6)	**<0.001**
SF-36-PF	77.4 (22.3)	52.7 (20.8)	**<0.001**
SF-36-RP	66.4 (41.0)	20.7 (31.1)	**<0.001**
SF-36-RE	77.6 (35.5)	38.3 (41.2)	**<0.001**
SF-36-VT	58.2 (17.8)	34.9 (15.2)	**<0.001**
SF-36-MH	68.5 (18.8)	54.2 (17.1)	**<0.001**
SF-36-SF	77.2 (19.5)	53.7 (17.3)	**<0.001**
SF-36-BP	64.7 (21.4)	37.7(14.5)	**<0.001**
SF-36-GH	54.3 (17.1)	27.6 (15.3)	**<0.001**
HADS-A	5.6 (3.3)	8.7 (3.6)	**<0.001**
HADS-A > 10	9.0 (15.5%)	22.0 (53.7%)	**<0.001**
HADS-D	4.7 (3.1%)	8.3 (3.7%)	**<0.001**
HADS-D > 10	7.0 (12.1%)	20.0 (48.8%)	**<0.001**
WPAI—Presenteeism	0.6 (1.7)	2.6 (3.0)	**<0.001**
WPAI—Work productivity loss	0.7 (1.7)	3.1 (3.3)	**<0.001**
WPAI—Limitation in non-work daily activities	1.8 (2.1)	5.4 (3.1)	**<0.001**

n (%); mean (SD). *p*-value calculated with Pearson’s Chi-squared test, Welch’s Two-Sample *t*-test, and Fischer’s exact test. Bold values indicate statistically significant *p*-values (*p* < 0.05).

**Table 6:** Prevalence of a CSI ≥ 40 in different cohorts of SpA patients.

Author	Year	N° Patients	Diagnosis	CSI ≥ 40
Guler [8]	2019	42	SpA	45.20%
Salaffi [20]	2024	157	PsA	45.20%
Kaya [18]	2023	35	AxSpA	41.00%
Sariyildiz [21]	2023	108	AxSpA	57.40%
Kieskamp [23]	2022	178	AxSpA	45%
Karlibel [6]	2023	82	AxSpA	45.10%

**Table 7:** Associations between CSI ≥ 40 and clinical characteristics of SpA patients.

Author	Psychiatric Disorders	Obesity	Enthesitis	Disease Activity	Female Sex
Salaffi [20]				*p* < 0.05	
Sariyildiz [21]	*p* < 0.05		*p* < 0.05		
Kieskamp [23]	*p* < 0.05	*p* < 0.05	*p* < 0.05		*p* < 0.05
Karlibel [6]				*p* < 0.05	*p* < 0.05

## Data Availability

The raw data supporting the conclusions of this article will be made available by the authors on request.

## Abbreviations

The following abbreviations are used in this manuscript:

ANOVA	Analysis of Variance
AS	Ankylosing Spondylitis
ASAS	Assessment of SpondyloArthritis International Society
ASDAS-CRP	Ankylosing Spondylitis Disease Activity Score with C-Reactive Protein
AxSpA	Axial Spondyloarthritis
bDMARDs	Biologic Disease-Modifying Anti-Rheumatic Drugs
BMI	Body Mass Index
CASPAR	ClASsification Criteria for Psoriatic Arthritis
COPD	Chronic Obstructive Pulmonary Disease
CNS	Central Nervous System
CRP	C-Reactive Protein
CS	Central Sensitization
CSI	Central Sensitization Inventory
csDMARDs	Conventional Synthetic Disease-Modifying Anti-Rheumatic Drugs
DAPSA	Disease Activity Index for Psoriatic Arthritis
FM	Fibromyalgia
FACIT-F	Functional Assessment of Chronic Illness Therapy–Fatigue
GCs	Glucocorticoids
HAQ	Health Assessment Questionnaire
HADS	Hospital Anxiety and Depression Scale
HADS-A	Hospital Anxiety and Depression Scale–Anxiety
HADS-D	Hospital Anxiety and Depression Scale–Depression
IBD	Inflammatory Bowel Disease
IASP	International Association for the Study of Pain
MRI	Magnetic Resonance Imaging
NSAIDs	Non-Steroidal Anti-Inflammatory Drugs
OA	Osteoarthritis
OP	Osteoporosis
PGA	Patient Global Assessment
PROMs	Patient-Reported Outcome Measures
PsA	Psoriatic Arthritis
QoL	Quality of Life
ReA	Reactive Arthritis
SF-36	Short Form-36 Health Survey
SpA	Spondyloarthritis
SpA-IBD	Spondyloarthritis associated with Inflammatory Bowel Disease
TMJD	Temporomandibular Joint Disorder
TTH	Tension-Type Headache
tsDMARDs	Targeted Synthetic Disease-Modifying Anti-Rheumatic Drugs
uSpA	Undifferentiated Spondyloarthritis
WPAI	Work Productivity and Activity Impairment Questionnaire
